# Premature Consolidation of Regenerated Bone During Limb Lengthening with External Fixation in Achondroplastic Patients: Case Series and Literature Review

**DOI:** 10.3390/jcm15176610

**Published:** 2026-08-27

**Authors:** Edoardo Verme, Luca Bianco Prevot, Domenico Curci, Eleonora Caboni, Fabio Verdoni

**Affiliations:** 1 Orthopedics and Traumatology Residency Program, University of Milan, Via Festa del Perdono 7, 20122 Milan, Italy; 2 IRCCS Istituto Ortopedico Galeazzi-Sant’Ambrogio, Via Cristina Belgioioso 173, 20157 Milan, Italy; luca.bianco96@gmail.com (L.B.P.); nicocurci73@gmail.com (D.C.); elecaboni@gmail.com (E.C.); fabio.verdoni@unimi.it (F.V.)

**Keywords:** achondroplasia, premature consolidation, premature consolidation, orthopedic pediatric, external fixation, limb lengthening, calloclasis

## Abstract

**Background**: Premature consolidation of the regenerated bone is a relevant and underreported complication of limb lengthening in achondroplasia, with a reported incidence of 7–15% in the international literature. The gain-of-function fibroblast growth factor receptor 3 (FGFR3) mutation paradoxically enhances bone-healing capacity, predisposing patients to accelerated callus maturation during distraction osteogenesis and exposing patients to a risk of invasive procedures to treat this issue. This study reports the incidence and clinical features of premature consolidation in a cohort of achondroplastic patients treated with external fixation. **Methods**: A retrospective analysis was conducted on patients with achondroplasia who underwent lower limb lengthening (femur and/or tibia–fibula) between 2017 and 2024 at IRCCS Ospedale Galeazzi–Sant’Ambrogio (Milan, Italy). Inclusion criteria were ages 7–18 years, confirmed achondroplasia, and a clinical–radiological diagnosis of premature consolidation. Key parameters included the healing index (HI), length gained, proportional increase, and fixator duration. **Results**: Among 39 patients (112 segments: 46 femora and 66 tibia–fibula units), premature consolidation occurred in 7 patients (10 segments), yielding an overall incidence of 8.9%. The fibula was predominantly affected (6/10 segments). The mean HI was 33.47 days/cm for the tibia and 30.1 days/cm for the femur; the mean length gained was 8.1 cm and 8.2 cm, respectively. **Conclusions**: Premature consolidation affects approximately 9% of lengthened segments in achondroplastic patients, with a predilection for the fibula due to its passive indirect distraction mechanism. Early radiographic recognition and dynamic adjustment of the distraction protocol are essential to prevent surgical calloclasis and optimize outcomes, and further analyses of the FGFR3 mutation are fundamental to understand the biologic features of the bone during the lengthening and consolidation steps.

## 1. Introduction

Achondroplasia represents the most common form of hereditary dwarfism and is characterized by a short stature and rhizomelic shortening of the limbs. It is a condition caused by an autosomal dominant mutation in the Fibroblast Growth Factor Receptor 3 (FGFR3) gene, which results in an alteration in the proliferation and differentiation of chondrocytes, leading to severely reduced longitudinal bone growth and a higher incidence of axial limb deformities [1,2].

A short stature and axial deformities of the lower limbs have a considerable impact on the quality of life of these patients, both in functional and psychological terms. One of the treatments of choice to improve the daily functions and quality of life of the patients is surgical limb lengthening. Throughout the decades, this technique has undergone a process of research and innovation to reach better results for the patients, reducing the risks and complications [1,3].

Lengthening procedures can be performed using devices such as telescopic nails or linear and circular external fixation systems. Limb lengthening with an external fixation system is extremely effective, allowing patients to increase the length of the treated bone segment by approximately 6–7 cm per surgical step; however, this surgery is not free from complications. The literature reports that the most frequent complications include infections, nerve injuries, muscle contractures, fractures of the regenerated bone, and premature consolidation of the regenerated bone [1,2,4].

The latter represents a clinically relevant complication, although it is less discussed than others, with a reported mean incidence ranging from 7% to 15%. It occurs when the patient’s regenerated bone forms too rapidly, interrupting the distraction step before lengthening has been completed. This premature consolidation leads to the inability to follow the lengthening program and the necessity to perform a corticotomy or calloclasis procedure to resume the lengthening process [1,2,5].

This complication has a multifactorial origin and can be influenced by both genetic conditions and mechanical factors related to the implant. Some patients, defined as rapid bone healers, are characterized by a rate of bone formation that exceeds the distraction rate set by the surgeon; alternatively, contributing factors may include suboptimal placement of the external fixator, incorrect osteotomy, or inadequate management of the lengthening process by the patient or the caregiver [1,2,4,6,7,8].

It is essential to identify the characteristics of patients who develop this complication and the factors associated with the surgical procedure in order to identify at-risk patients early, follow them with dedicated postoperative monitoring, and apply a lengthening protocol designed to prevent premature ossification of the regenerated bone [9].

This study describes the rate of premature consolidation of regenerated bone observed in the case series of a referral center for the treatment of achondroplastic patients, the characteristics of patients with external fixation undergoing limb lengthening who developed this complication, and a critical analysis of the literature on premature consolidation of the regenerated bone.

## 2. Materials and Methods

This study retrospectively analyzed patients affected by achondroplasia who underwent lower limb lengthening procedures (femur and tibia + fibula) between 2017 and 2024 at the Pediatric Orthopedics Division of IRCCS Ospedale Galeazzi–Sant’Ambrogio (Milan, Italy). The database was retrospectively constructed by reviewing the clinical documentation (medical records, radiographs, and outpatient reports) of patients with achondroplasia. The inclusion criteria were as follows: patients between 7 and 18 years old with a diagnosis of achondroplasia who were treated with lengthening using a linear external fixator (femoral lengthening) or a circular external fixator (tibial lengthening) and a clinical and radiological diagnosis of premature consolidation of the regenerated bone. The exclusion criteria were administration of vosoritide during the lengthening process, ages below 7 years or above 18 years, and other forms of skeletal dysplasia. The diagnosis of achondroplasia had been established previously by specialists for all patients who presented to our institution for limb lengthening. The femur was considered a single segment; the tibia and fibula were considered a single segment. For each patient the following parameters were evaluated: the lengthening step; the length of the femur and that of the tibia and fibula before surgery (cm); the length of the femur and that of the tibia and fibula after surgery (cm); the length gained after lengthening (cm); the proportional increase in limb length relative to total length; the healing index (HI), which was calculated as the ratio between the total number of days the fixator was maintained and the total centimeters gained; the total number of days the fixator was maintained (days); age; and sex. Patients underwent bilateral tibial and fibular lengthening with an RRS ring fixator by DialMedicali (Milan, Italy). For bilateral femoral lengthening, the axial external fixator by DialMedicali (Milan, Italy) was used.

The lengthening protocol followed in this study is based on the validated format described in previous studies such as Peretti et al. and is described in 4 steps: steps 1 and 3 encompass tibial and fibular lengthening and steps 2 and 4 involve femoral lengthening [1].

The first surgical step for tibial and fibular lengthening, which is performed under fluoroscopic guidance, consists of a fibular osteotomy at the distal third. The proximal and distal tibiofibular joints were each transfixed with a Kirschner wire (K-wire) to prevent distal or proximal migration of the fibula, respectively. The proximal ring of the fixator was then positioned parallel to the knee joint line and secured with 2 K-wires and 2 half-pins with a diameter of 6.0 mm diameter. Subsequently, the distal ring was positioned and anchored to the bone with 2 half-pins with a diameter of 5.0 mm. The two rings were connected with four lengthening bars. A tibial osteotomy was then performed below the tibial tuberosity.

The first surgical step for femoral lengthening consists of the placement of 2 half-pins of 6.0 mm at the proximal end and 2 half-pins of 6.0 mm at the distal end of the femur, perpendicular to the anatomical axis. In all cases, a transverse osteotomy was performed in the proximal metaphyseal region. All procedures were conducted under fluoroscopic guidance and performed by the same surgeon.

Following surgery, weight bearing was permitted on postoperative day 2 or 3 with the aid of a walker. Patients underwent a physiotherapy program for range-of-motion (ROM) recovery, followed by maintenance of the ROM of the ankle, knee, and hip joints for at least 1 year.

On the third postoperative day, the lengthening phase was initiated at a rate of 1.0 mm/day for the first 10 days; thereafter, distraction was distributed throughout the day (0.5 mm every 12 h).

Patients were assessed clinically and radiologically every 2 weeks during the lengthening period and monthly during the consolidation period.

Premature consolidation was diagnosed based on physical examination and radiological assessment as fusion or corticalization of the regenerated bone, with the inability to continue lengthening and severe pain reported by the patient during attempted distraction.

The incidence of premature consolidation was calculated as the ratio between the number of segments developing premature consolidation and the total number of segments.

Informed consent was obtained from all patients (from parents or legal guardians in the case of minors); the observational nature of the study meant that it did not require ethics committee approval.

During the preparation of this manuscript/study, the authors used Claude Sonnet 5 for the purposes of improving the English language and sentence correction. The authors have reviewed and edited the output and take full responsibility for the content of this publication.

## 3. Results

Data from 39 patients who underwent lower limb lengthening were analyzed, for a total of 112 lengthened segments: 46 femurs and 66 tibiae with fibulae.

At the conclusion of the analysis, seven patients developed premature consolidation, involving a total of 10 segments: 4 femurs (bilaterally) and 6 fibulae (4 unilateral and 1 bilateral).

The cohort consisted of three males and four females with a mean age of 10.6 years (range: 7.0–13.0 years), a mean weight of 27.7 kg (range: 21.0–40.0 kg), and a mean height at the time of surgery of 114.3 cm (range: 97.0–142.0 cm). In three patients, premature consolidation occurred bilaterally (one fibula and two femurs).

All patients were in good general health and presented no comorbidities.

Premature consolidation affected both femurs in two cases, the right fibula in two cases, the left fibula in two cases, and both fibulae in one case. Two patients were at the second femoral lengthening step, three patients were at the first tibial lengthening step, and two patients were at the second tibial lengthening step.

**Figure 1:**
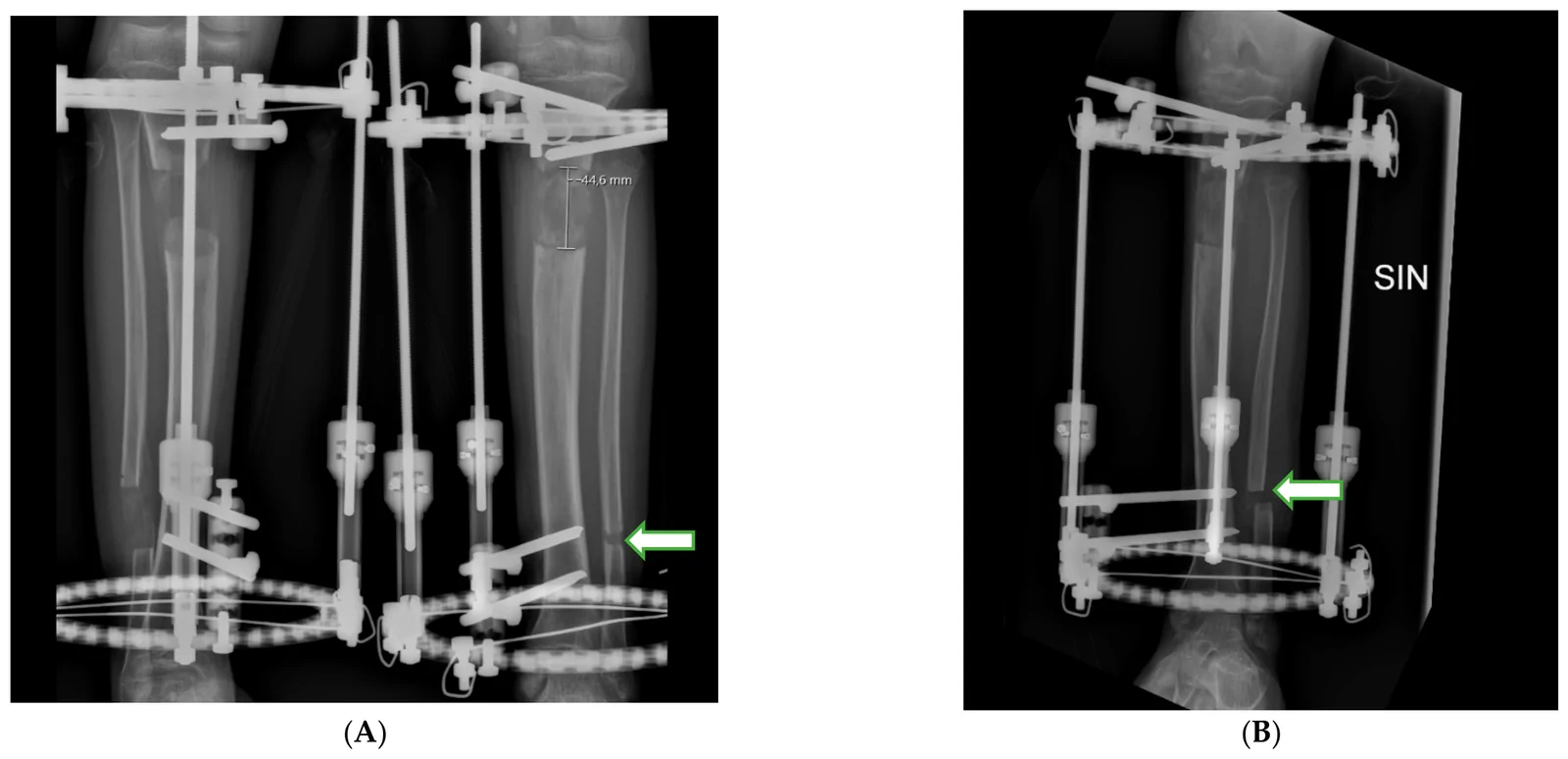
(**A**) X-ray showing premature consolidation of the left fibula compared to the contralateral, as indicated by the white arrow. (**B**) X-ray showing the outcome of the calloclasis on the left fibula of the patient, as indicated by the white arrow.

Patients with premature consolidation (Figure 1A,B) included in this study had a mean preoperative tibial length of 18.9 cm (range: 14.6–27.4 cm), reaching a mean tibial length at the end of the lengthening process of 27.0 cm (range: 20.5–36.6 cm).

The mean length gained by the tibia during lengthening was 8.1 cm (range: 5.5–9.3 cm); the mean proportional increase was 44.1% (range: 37.0–55.0%), and the mean HI was 33.4 days/cm (range: 6.4–45.7 days/cm). The mean duration of the period during which patients maintained the external fixator was 267.8 days (range: 194.0–420.0 days).

**Figure 2:**
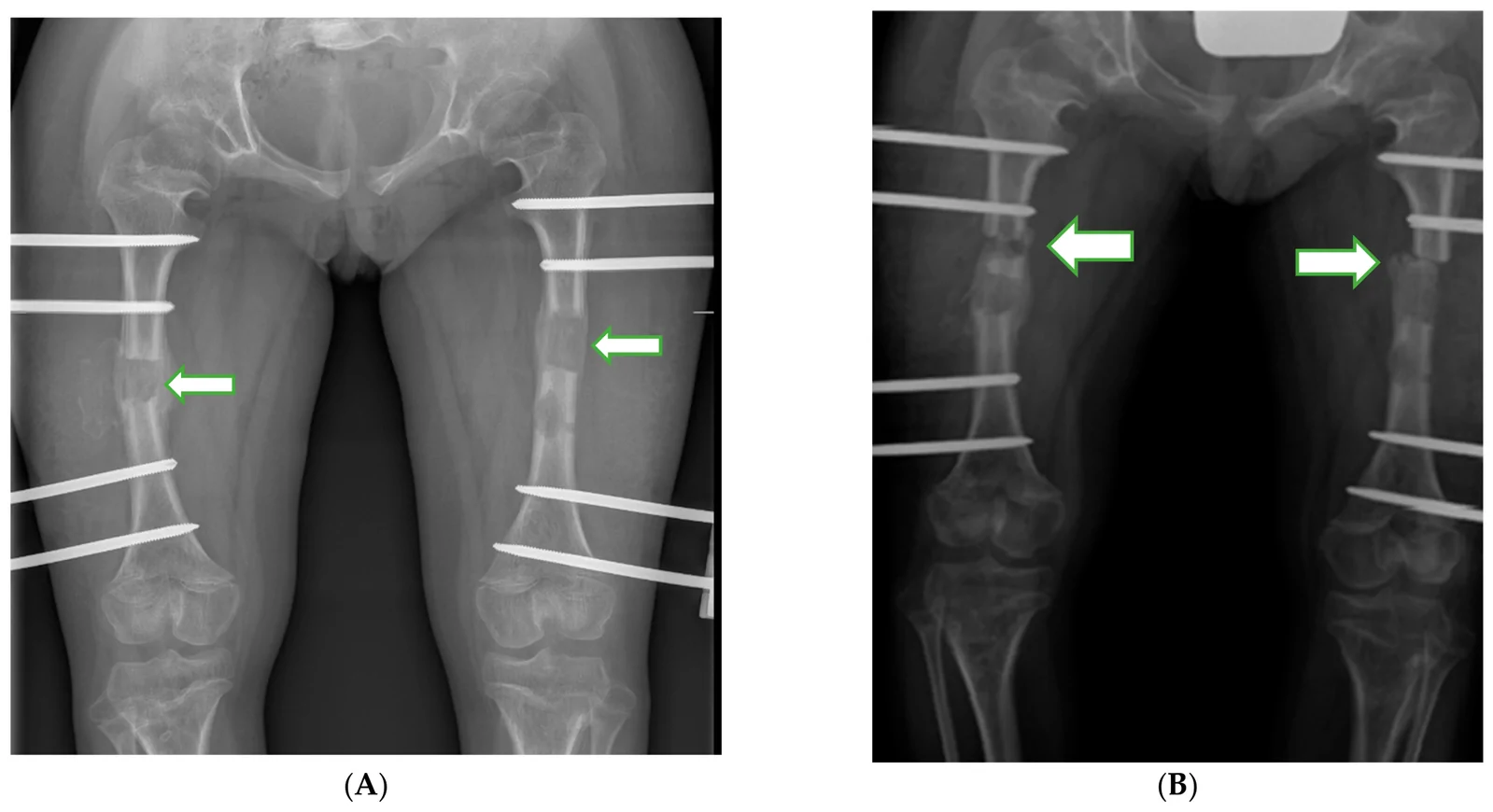
(**A**) X-ray showing premature consolidation of femurs bilaterally, as indicated by the white arrow. (**B**) X-ray showing the outcome of the calloclasis on the femurs bilaterally, as indicated by the white arrow.

Regarding patients with premature consolidation at the femoral level (Figure 2A,B), the mean preoperative femoral length was 17.9 cm (range: 17.4–18.0 cm), reaching a mean femoral length at the end of the lengthening process of 26.0 cm (range: 25.0–26.9 cm).

The mean femoral length gained during lengthening was 8.2 cm (range: 7.0–9.5 cm); the mean proportional increase was 46.0% (range: 39.0–54.0%), and the mean HI was 30.1 days/cm (range: 28.7–31.6 days/cm).

The mean duration of the period during which patients maintained the external fixator was 247.0 days (range: 221.0–271.0 days).

The mean interval between initial external fixator implantation and the corticotomy/calloclasis procedure for the prematurely consolidated regenerate was 53.4 days (range: 40.0–67.0 days) for the tibia and fibula and 51.0 days (range: 46.0–56.0 days) for the femur. The mean interval between corticotomy/calloclasis and external fixator removal was 213.4 days (range: 154.0–346.0 days) for the tibia and fibula and 196.0 days (range: 175.0–217.0 days) for the femur.

An overall premature consolidation rate of 8.9% was identified (the ratio of segments with premature consolidation to total lengthened segments), a figure that was identical when calculated separately for the tibia–fibula group and the femoral group.

## 4. Discussion

In our series the overall incidence of premature consolidation of the regenerate was 8.9% when calculated across all lengthened segments, with a comparable distribution between tibio-fibular and femoral sites. This figure falls within the 7.0–15.0% range reported in the external-fixator literature and confirms that the complication is clinically relevant and far from rare in the achondroplastic population [1,5,10]. Several factors, both patient-related and device-related, concur in determining the risk of premature consolidation, and each of them deserves a separate consideration.

The first and most distinctive factor is the biology of the achondroplastic bone itself. The gain-of-function mutation in FGFR3, the molecular hallmark of the disease, inhibits chondrocyte proliferation and differentiation in the proliferative zone of the growth plate, dramatically reducing longitudinal bone growth; at the same time, paradoxically, the very same mutation enhances the bone-healing response to mechanical stimulation [11]. The achondroplastic bone grows less but heals fast, and during distraction osteogenesis this translates into a regenerate that can mature and corticalize before the planned length is achieved. Experimental and molecular data converge on this point [12]. In a murine model of distraction osteogenesis, Osawa et al. showed that Fgfr3*ach* transgenic mice form calluses significantly faster than wild-type controls, with accelerated endochondral ossification already evident at days 4.0–7.0 after the end of the distraction phase [13]. The mechanism is paracrine as much as chondrogenic: FGFR3 activation upregulates BMP ligands; downregulates their antagonists in the perichondrium, enhancing local osteoblast differentiation; and alters osteoblastogenesis and osteoclastogenesis via p38 signaling, accelerating remodeling at the osteotomy site. The clinical consequence is straightforward: the same mutation that impairs longitudinal growth enhances the healing response, shortening bone regenerate maturation and increasing the risk of premature consolidation.

The second factor is the device used, which in our series, as in most pediatric and adolescent achondroplastic cohorts, is the external fixator (Ilizarov circular and monolateral). The external fixator is widely used in this population for several reasons: the patients are usually skeletally immature, with narrow medullary canals and short bones that do not accommodate intramedullary nails of adequate length; the procedure is bifocal or multilevel in a substantial proportion of cases; and deformity correction in the coronal and sagittal plane, frequently needed in these patients, is controllable with a circular frame. The trade-off is well known and includes pin-tract infection, joint stiffness, prolonged frame time and a longer healing index.

A useful term of comparison is the motorized intramedullary lengthening nail (PRECICE^®^ and its successors), which is increasingly used in skeletally mature patients. The mean healing index reported with this device in achondroplasia is markedly lower than that reported with external fixators (around 20.0 days/cm vs. 30.0–40.0 days/cm), and Vogt et al. observed accelerated consolidation requiring an upward adjustment of the distraction rate in 33% of treated segments, confirming that, regardless of the device, the achondroplastic regenerate matures faster than the protocol predicts [14].

Closely linked to the device is the third factor: the distraction protocol, which cannot be a fixed parameter but must be tailored to the patient and adjusted dynamically during follow-up. Our standard protocol was 1.0 mm/day for the first 10 days, then 0.5 mm every 12 h during the active phase, in line with the most widely accepted scheme. The mean HI was 33.5 days/cm at the tibia and 30.1 days/cm at the femur, values at the lower end of the published range for external fixators. Vaidya et al. reported a mean tibial HI of 26.1 days/cm with the Ilizarov fixator; Park et al. described a striking site-specific difference in 28 achondroplastic patients (tibial HI: 10.7 days/cm vs. femoral HI: 28.1 days/cm), with the femur consistently showing longer regenerate healing times [5]. The meta-analysis by Hosny et al., including 14 studies and 1,149 achondroplastic patients, sets the mean HI across the literature at 37.1 days/cm (95% CI: 32.3–41.8), with an overall complication rate of 56.1% (95% CI: 26.9–85.2%) and a mean fixation duration of 7.7 months [10]. Reported HI values range from 20.0 to 63.0 days/cm: this wide variability indicates that, even within the achondroplastic population, individual healing kinetics differ enough to make a single fixed protocol unsuitable. Park et al. addressed this with an adaptive approach, modifying the distraction rate according to the morphological callus pattern described by Li et al. and reassessing it at biweekly intervals [15,16]. Vogt et al. increased the rate in 5 of 15 nail-treated patients (33.0%) at the first sign of accelerated consolidation, avoiding corticotomy [14]. As for the frequency of daily distraction, Bright et al. compared high-frequency motorized distraction (1/1440 mm delivered 14,440 times per day) with conventional manual distraction (0.25 mm four times per day) and found no significant differences in consolidation times or complications, suggesting that delivery frequency is not the determining factor. From a clinical standpoint, these data highlight the need for close monitoring throughout the active phase, along with biweekly radiographic follow-up and careful evaluation of the callus morphology. When signs of accelerated consolidation are observed, such as early dense filling of the distraction gap, loss of the typical fusiform appearance, or early appearance of continuous cortices, an upward adjustment of the distraction rate can prevent premature consolidation and avoid the need for surgical calloclasis. Increased resistance felt during distraction of the fixator is a further sign worth monitoring, as it may indicate early bridging of the regenerate and should prompt reassessment of the rate. This option, however, must always be weighed against the tolerance of the surrounding soft tissues, which cannot be lengthened all at once: an excessively rapid rate outpaces the adaptive capacity of muscles and tendons, leading to contractures. In our experience, increases must therefore be cautious, accompanied by intensive physiotherapy, and discontinued at the first signs of soft-tissue suffering [4,17,18]. A distinctive finding of our series is the frequent involvement of the fibula in premature consolidation, which was observed in 6 of the 10 affected segments. A possible explanation is that the fibula, which was fixed only proximally and distally with a single Kirschner wire rather than directly engaged by the distraction mechanism, may receive a weaker traction force than the tibia, which is transmitted passively through the interosseous membrane and surrounding soft tissues. This reduced force could translate into a less effective distraction at the fibular osteotomy site, potentially making it insufficient to keep pace with the fast healing kinetics of the achondroplastic bone. This interpretation remains hypothetical and would require dedicated studies to confirm. Mindler et al. [2] noted that surgical strategies to restore tibiofibular alignment still lack robust evidence and remain an open area of research. The diagnosis of premature consolidation remains essentially clinical and radiographic, relying on three main parameters, namely the Pixel Value Ratio (PVR), the morphological callus classification and the corticalization status. The PVR offers a quantitative estimate of regenerate mineralization; Park et al. reported a mean PVR at 28 weeks of 1.2 at the tibia and 1.0 at the femur, with values close to or above 1 indicating corticalization [15]. The PVR is informative but lacks inter-institutional standardization and depends on acquisition conditions, limiting its routine applicability. The morphological callus classification of Li et al., which was later adopted by Park et al., identifies a cylindrical or fusiform callus with homogeneous density as physiological, whereas early dense filling of the distraction gap is the most reliable warning sign of incipient premature consolidation [15,16]. Early corticalization, defined as three or more continuous cortices on anteroposterior and lateral views during the active phase, is the most widely accepted definitive radiographic criterion, as used by Verdoni et al. and Vaidya et al. These parameters complement, but do not replace, clinical judgment when deciding whether to accelerate distraction, intensify follow-up or proceed to calloclasis. Premature consolidation should be viewed not only as a complication to manage but also as an individual biological marker, whose early appearance signals above-average healing capacity and calls for real-time protocol adjustment. Paley, in his series of 75 achondroplastic patients with a mean lengthening value of 27.0 cm, showed that excellent long-term outcomes depend on dynamic, individualized management. Liantis et al. identified the healing index and adult age as the main predictors of distraction-related complications, confirming bone-healing kinetics as the principal driver of overall complication risk [4,7]. Several technical questions remain open: whether osteotomy technique (percutaneous vs. open, along with periosteal preservation) affects consolidation speed; how fixator configuration influences callus quality; and whether differences in healing index values between external fixators and intramedullary nails translate into meaningfully different rates of premature consolidation. Across these open questions, our experience consistently points to the need for a high degree of individualization in limb lengthening for achondroplasia, balancing bone-healing kinetics against soft-tissue tolerance.

## 5. Conclusions

The limitations of this study include the small sample size of patients with premature consolidation, the monocentric nature of the cohort subjected to selection bias, the absence of a comparison group of achondroplastic patients without premature consolidation to evaluate which parameters differ, the impossibility of performing qualitative histological assessments of the premature consolidation zones following calloclasis, and the absence of standardized outcomes (particularly regarding complications, fixation duration, HI, and regenerate quality) that could be examined in future studies. To date, there are no prospective randomized studies comparing different osteotomy strategies or fixator configurations specifically in the achondroplastic population, a gap that represents the primary limitation of the current literature and that future research must address.

## Data Availability

The data supporting the findings of this study are available from the corresponding author upon request.
